# Non-Invasive Magnetic Resonance Imaging in Rats for Prediction of the Fate of Grafted Kidneys from Cardiac Death Donors

**DOI:** 10.1371/journal.pone.0063573

**Published:** 2013-05-07

**Authors:** Jun-Ya Kaimori, Satomi Iwai, Masaki Hatanaka, Takumi Teratani, Yoshitsugu Obi, Hidetoshi Tsuda, Yoshitaka Isaka, Takashi Yokawa, Kagayaki Kuroda, Naotsugu Ichimaru, Masayoshi Okumi, Koji Yazawa, Hiromi Rakugi, Norio Nonomura, Shiro Takahara, Eiji Kobayashi

**Affiliations:** 1 Department of Advanced Technology for Transplantation, Osaka University Graduate School of Medicine, Osaka, Japan; 2 Laboratory of Small Animal Surgery I, School of Veterinary Medicine, Kitasato University, Aomori, Japan; 3 Department of Geriatrics and Nephrology, Osaka University Graduate School of Medicine, Osaka, Japan; 4 Center for Development of Advanced Medical Technology, Jichi Medical University, Tochigi, Japan; 5 BioView, Tokyo, Japan; 6 Tokai University School of Information Science and Technology Department of Human and Information Science, Kanagawa, Japan; 7 Department of Specific Organ Regulation(Urology), Osaka University Graduate School of Medicine, Osaka, Japan; University of Sao Paulo Medical School, Brazil

## Abstract

The main objective of this study was to assess cardiac death (CD) kidney grafts before transplantation to determine whether blood oxygen level-dependent (BOLD) and diffusion MRI techniques can predict damage to these grafts after transplantation. We assessed CD kidney tissue by BOLD and diffusion MRI. We also examined pathological and gene expression changes in CD kidney grafts before and after transplantation. Although there was significantly more red cell congestion (RCC) in the inner stripe of the outer medulla (IS) in both 1 h after cardiac death (CD1h) and CD2h kidneys destined for grafts before transplantation compared with CD0h (p<0.05), CD2h, but not CD1h, kidney grafts had significantly different RCC in the IS 2 days after transplantation (p<0.05). Consistent with these pathological findings, tissue plasminogen activator (tPA) gene expression was increased only in the cortex and medulla of CD2h kidney grafts after transplantation. BOLD MRI successfully and non-invasively imaged and quantified RCC in the IS in both CD1h and CD2h kidney grafts (p<0.05). Diffusion MRI also non-invasively assessed increased the apparent diffusion coefficient in the IS and decreased it in the outer stripe (OS) of CD2h grafts, in concordance with interstitial edema in the IS and tubule cellular edema in the OS. These two types of edema in the outer medulla could explain the prolonged RCC in the IS only of CD2h kidney grafts, creating part of a vicious cycle inhibiting red cells coming out of capillary vessels in the IS. Perfusion with University of Wisconsin solution before MRI measurements did not diminish the difference in tissue damage between CD1h and CD2h kidney grafts. BOLD and diffusion MRI, which are readily available non-invasive tools for evaluating CD kidney grafts tissue damage, can predict prolonged organ damage, and therefore the outcome, of transplanted CD kidney grafts.

## Introduction

The worldwide shortage of organs for transplantation has highlighted expanded criteria donors (ECDs) and donation after cardiac death (DCD) as new organ sources. Because kidney grafts from ECDs and DCD are more susceptible to additional ischemia-reperfusion injuries than are those from living or brain-dead donors, these grafts are more likely to develop primary dysfunction and delayed graft loss[1]. In spite of considerable efforts to protect such marginal donor kidneys, conventional static cold storage combined with intra-cellular type solution has remained the primary option, even for damaged kidneys [2]. However, recent evidence has shown that the use of normothermic recirculation (NR) has protective effects on ECD and DCD organs. Most of this evidence has focused on NR prior to DCD liver transplantation [3]–[5]. In the case of renal transplantation, one clinical study reported significant improvements in delayed graft function and graft survival after transplantation using NR compared with conventional organ cooling methods [6]. One of the common features of ischemic kidney damage, congestion of blood in the capillaries of the inner stripe(IS) of the outer medulla, is thought to possibly impair both circulation and tubular flow [7]. Although its pathogenesis is still obscure, it is proposed that dramatic capillary permeability changes in the IS cause trapping of erythrocytes, which then accumulate and ultimately plug the circulatory system [8], [9]. Furthermore, the coagulation system is not involved in this type of congestion, which is known as “red cell trapping”, or “red cell congestion (RCC)”, because the anti-coagulants acetylsalicylic acid and heparin reportedly do not improve renal failure or congestion in the medulla [7].

To improve graft function, non-invasive tools for assessing tissue damage in grafts are required. In the process of seeking optimal storage conditions for DCD kidney grafts, we found that bilaterally nephrectomized rats transplanted with cardiac death (CD) 0 and 1 h kidneys survived. However, rats with CD2h kidneys that were otherwise subjected to the same conditions died within 5 days with increased serum creatinine and urea nitrogen concentrations, suggesting they had died of renal failure [10]. Preliminary tissue examination of the transplanted CD kidneys revealed that, although both CD1h and CD2h kidney grafts had congested medullas before transplantation, only CD2h kidneys had prolonged RCC in their medullas post-transplantation; CD0h and CD1h kidneys did not. Thus, detection of sustained RCC in the medulla can predict graft function.

Because renal ischemia causes congestion of the IS of the outer medulla characterized by massive erythrocyte accumulation and dramatic movement of fluid, we used blood oxygen level-dependent (BOLD) and diffusion-weighted MRI techniques to investigate these phenomena non-invasively. The BOLD MRI technique, which has chiefly been used in the field of brain science [11], relies on the fact that oxygenated and deoxygenated forms of hemoglobin differ in their magnetic characteristics. This affects the neighboring magnetic fields, leading to changes in the T_2_* relaxation time of adjacent water molecules. Such changes influence the signal intensities in T_2_* weighted MRI [12]. Therefore, this technique would permit non-invasive evaluation of RCC in the medullas of CD kidney grafts. Another MRI technique, diffusion MRI, can detect molecular diffusion because, under a magnetic field gradient, random molecular motion such as Brownian motion causes incoherent phase shifts, which lead to attenuated signals[13], [14]. By using diffusion-weighted (DW) MRI data, it is possible to calculate an apparent diffusion coefficient (ADC), which represents microscopic translational motion, including both molecular diffusion of water and microcirculation of blood in capillary perfusion [14]–[16]. Therefore, the ADC provides valuable information about the structure of tissues such as interstitial edema, cellular edema [17], cellar atrophy [17], and fibrosis [18]. Therefore, we used diffusion-weighted MRI to non-invasively evaluate fluid distribution changes in CD kidney graft tissue.

In the present study, we used these two non-invasive MRI techniques to evaluate CD kidney grafts tissue before transplantation. We found that BOLD MRI was able to detect RCC in the IS of the outer medulla. DW MRI revealed that ADC values were decreased in the outer stripe (OS) but increased in the IS in CD2h kidneys compared with CD 0h kidney grafts. These data might elucidate the pathophysiology of ischemia-induced blood congestion of the medulla. Conventional perfusion with University of Wisconsin (UW) solution partially washes RCC out of the IS of CD1h kidney grafts, but not out of CD2h kidney grafts. At the same time, the interstitial tissues of perfused CD2h kidney cortexes (CTX), OS, and IS become massively edematous. Thus, the process of perfusion with UW solution would increase the difference between damage to CD1h and CD2h kidney grafts. BOLD and DW MRI can detect these changes. MRI data may make it possible to predict prolonged RCC in the medullas of transplanted CD kidney grafts, leading to accurate assessment of graft outcomes before transplantation.

## Materials and Methods

### Animals

The present experiments were performed in three places: the Institute of Experimental Animal Sciences of the Osaka University Medical School (Osaka, Japan), the Business Support Center for Biomedical Research Activities (Kobe, Japan), and Kitasato University (Aomori, Japan). Male wild LEW rats weighing 250–300 g were purchased from Japan SLC (Breeding Laboratories, Shizuoka, Japan) by Osaka University, from Oriental Bioservice (Kyoto, Japan) by the Business Support Center for Biomedical Research Activities, and from Charles River (Breeding Laboratories, Kanagawa, Japan) by Kitasato University. Rats in these three facilities were kept in strictly controlled environments with fixed temperature and humidity, 12 h light/dark cycles and were provided with standard laboratory chow and water *ad libitum*. All experiments were conducted according to established guidelines for animal welfare and approved by the Animal Ethics Committees of Kitasato (09-149) and Osaka Universities (22-057-0).

### Transplantation procedure

Rat kidney transplantations were performed as described previously [10] at Kitasato University. After being killed, donor LEW rats were kept in a room at 23°C for 0, 1, or 2 h before the left kidney was harvested. The harvested kidneys, which were not processed with any preservation solutions, were transplanted into bilaterally nephrectomized recipient LEW rats immediately after being excised from the donor bodies. Two days after transplantation, recipient rats were killed and the transplanted kidneys harvested, half of each kidney being processed for pathologic evaluation and the other half processed for assessment of gene expression.

### Assessment of gene expression

One half of each harvested kidney was dissected into cortex and medulla and each of the tissue samples homogenized by a Polytron tissue homogenizer PT 1300D (Kinematica AG, Luzern, Switzerland). mRNA was extracted with TRIzol solution (Life Technologies, Carlsbad, CA, USA). The extracted mRNA was converted into cDNA using a PrimeScript reverse transcription polymerase chain reaction (RT-PCR) kit (Takara, Shiga, Japan). Real-time PCR was performed with SYBR Premix Ex Taq (Takara) according to the manufacturer’s manual and with a Thermal Cycler Dice Real Time System Single (TP850) (Takara). The real-time PCR data were processed and evaluated with unpaired t-test by Multiplate RQ software (Takara). The gene expression data were normalized by beta-actin. The primer sequences for real-time PCR of rat heme oxygenase 1 (HO-1), 70 kilodalton heat shock protein (HSP-70), interleukin- 6 (IL-6), kidney injury molecule 1 (KIM-1), transforming growth factor beta (TGF-β), α-smooth muscle actin (α-SMA), hypoxia-inducible factor 1-alpha (HIF-1α), and monocyte chemoattractant protein-1 (MCP-1) were those reported by Moers et al. [19]. The primer sequences for real-time PCR of rat tPA, urokinase (uPA), urokinase receptor (uPAR), and plasminogen activator inhibitor-1 (PAI1) were as follows: tPA, sense 5′-GGC GAA ATG GGA TGA AGG T-3′, antisense 5′-GGT GGT ATA GTT CCC TGC CTT AAA-3′; uPA, sense 5′-GGG GAA GTC CTA TAA CCC GG-3′, antisense 5′-TGG AAG GCC AGA GTT TCG TC-3′; uPAR, sense 5′-AGG GAG GGA GTT TTG AGG TG-3′, antisense 5′-CCG GAG CCC TTT TCT CAA TTC-3′; PAI-1, sense 5′-ccr CGG TGG TGG GTA TGG T-3′, antisense 5′-GTG CCC GTG TGA CTG ATA TTG AA-3′.

The quantification of gene expression was performed to detect cytoprotection (HO-1 and HSP-70), tissue regeneration and fibrosis (TGF-b), renal tubular injury (KIM-1), inflammation (IL-6, HIF-1a, and MCP-1), coagulation (tPA, uPA,and uPAR) and interstitial fibrosis (a-SMA) 24 h after transplantation[19].

### Measurements of areas of trapped red cell

Half of each harvested kidney was fixed with 4% paraformaldehyde phosphate-buffered saline and embedded in paraffin. Two-micron paraffin-embedded sections were stained with eosin. Trapped red cell areas in cortex, outer strip, inner stripe of outer medulla, and inner medulla were measured with Image J (National Institute of Health, Bethesda, MD, USA).

### Perfusion of CD kidney grafts with UW solution

After being killed, donor LEW rats were kept in a room at 23°C for 0, 1, or 2 h before the left kidney was perfused. The perfusion catheter was placed in the inferior vena cava just below the renal artery. The renal vein was cut and the inferior vena cava closed just above the renal artery. Perfusion with UW solution was performed by 1.5 mL UW solution injected through a syringe and subsequently by drip (1.5 mL) from a 75 cm height. All perfusion processes took within 4 minutes.

### MRI measurement of CD kidney grafts in UW solution

**Figure 1:**
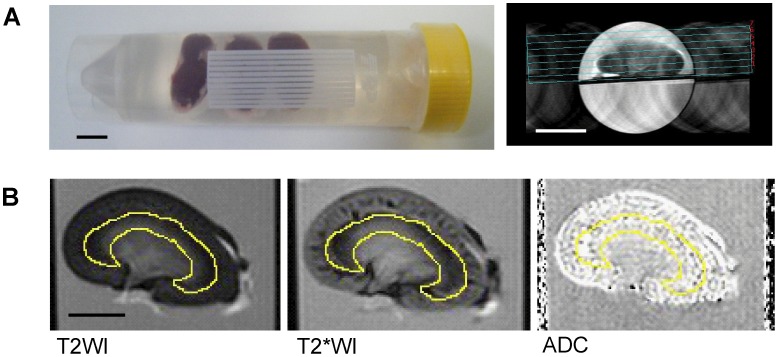
MRI measurement methods for cardiac death (CD) kidney grafts. (A) Left: Harvested kidneys from CD donors soaking in University of Wisconsin (UW) solution. Three left kidneys were attached to plastic plates and soaked in a 4°C UW solution immediately after harvest or perfusion. Scale bar: 1 cm. Right: The sectional positions of longitudinal kidney MRI images. To get the mid longitudinal plane image of a kidney graft, series of MRI images were taken in parallel with the plastic plate. Scale bar: 1 cm. (B) MRI images of selected regions referenced by T_2_ weighted images. T_2_* signal intensity (SI) and apparent diffusion coefficient (ADC) were measured in the same regions referenced T2weighted images by Image J software. Scale bar: 0.5 cm.

All MRI images were obtained on a Unity INOVA MR spectrometer (Varian Associates, Palo Alto, CA, USA) with a JASTEC Horizontal Magnet 4.7T (JMTB-4.7/310/SS) (Japan Superconductor Technology, Hyogo, Japan). A Varian volume coil was used for radiofrequency excitation and signal detection. Three harvested left kidneys were attached to plastic plates with instant glue and soaked in a 4°C UW solution (ViaSpan®, Astellas Pharma, Tokyo, Japan) in a 50 mL tube (Figure 1A) as soon as they had been removed from the donor rats at the specified time after CD with or without perfusion process. The MRIs were performed immediately after removal of the kidneys and 3 h incubation in 4°C UW solution. Each measurement was conducted in a room at 13°C and took about 1 h. During each measurement, the kidney temperature was kept at about 8°C. To keep the different imaging sequences comparable, all images were acquired with the same longitudinal planes (Figure 1A). An image matrix of 256×128 and field of view (FOV) of 100 mm×50 mm resulted in a nominal pixel size of 0.39 mm×0.39 mm.

### BOLD MRI

To achieve relatively high temporal resolution (256×128) with BOLD contrast, we used a gradient recalled echo pulse sequence without spoiler gradients to enhance the contrast made by the T_2_* changes. The MRI parameters were as follows: repetition time (TR)  = 30 ms, echo time (TE)  = 10 ms, flip angle  = 13°, image acquisition matrix  = 256×128, FOV  = 100×50 mm, slice thickness  = 1 mm, and two averages.

### Diffusion MRI

To obtain an ADC map, diffusion-weighted spin-echo pulse sequences with gradient strength corresponding to b value (diffusion sensitivities) of 0,134 (s/mm^2^) were used. The MRI parameters were as follows: TR  = 2 s, TE  = 35 ms, image matrix  = 256×128, FOV  = 100×50 mm, slice thickness  = 1 mm, and two averages. ADC was calculated according to the following equation:

ADC  =  (ln [S_1_+S_h_])/(b_h_–b_1_) where ln refers to the natural logarithm, and S_1_ and S_b_ denote signal intensities of 0 and 134 s/mm^2^ b value, respectively.

### Measurement of T_2_* signal intensity and ADC in four regions of kidney tissue

To accurately measure T_2_* signal intensity and ADC in four regions (CTX, OS, IS, and inner medulla [IM]), Image J software and its restore selection function were used. This function was used to ensure selection of exactly the same region of kidney that had been anatomically referenced in the standard T_2_ weighted image for measurement of T_2_* signal intensity and ADC (Figure 1B).

### Statistics

An unpaired t-test with equal variance was used to compare two different kidney tissues and a paired t-test with equal variance for tests within the same kidney tissues. When more than three different kidney tissues were compared, one-way ANOVA and Tukey’s multiple comparison test were used. All tests were conducted with GraphPad Prism 5 software (GraphPad Software, CA, USA). In tables, data were shown in means ± standard deviation.

## Results

### Red blood trapping in transplanted kidneys

**Figure 2:**
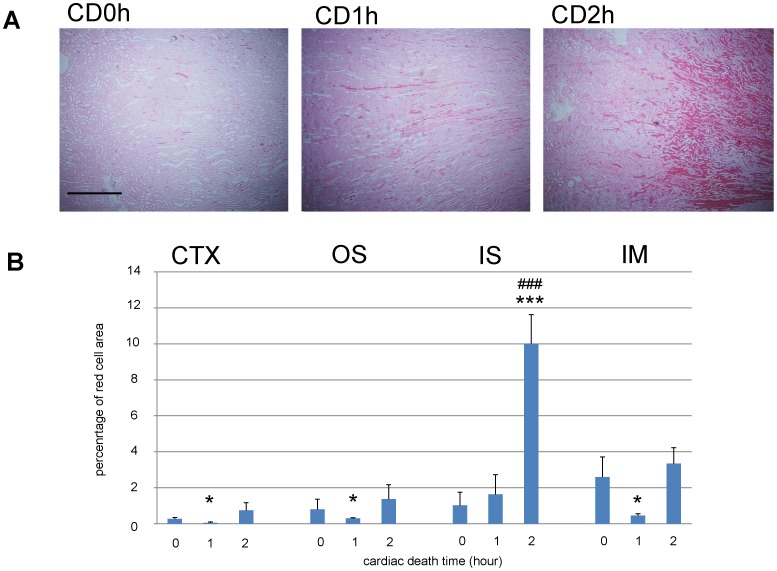
Massive red cell congestion (RCC) was observed only in inner stripe of the outer medulla (IS) of 2h cardiac death (CD2h) transplanted kidney grafts. (A) Eosin-stained photo micrographs of regions of transplanted kidney grafts. These depict the transition area between the outer and inner stripes of the outer medulla. Massive RCC was seen in IS of CD2h kidney graft. Scale bar: 0.5 mm. (B) Quantification of areas of RCC in various regions of CD kidney grafts. In IS of CD2h kidneys, RCC culminated in massive quantities compared with CD0h or CD1h. *, p<0.05 vs CD0h; ***, p<0.001 vs CD0h unpaired t-test; ###, p<0.001, one-way ANOVA and Tukey’s test.

Previously, we reported that kidney grafts from 2hr cardiac death donors (CD2h) failed to function properly, as evidenced by increased blood urea nitrogen (BUN) and creatinine concentrations, and that these rats did not survive beyond 5 days after transplantation [10]. To investigate the cause of graft loss of transplanted CD2h kidneys, we examined kidney specimens from CD0h, CD1h, or CD2h 2 days after transplantation. As previously reported [20], transplanted kidneys from CD0h, CD1h, or CD2h produce equal degrees of red cell congestion (RCC) in the IS 1 h after transplantation. However, we found that RCC persisted only in transplanted CD2h kidneys and not in CD0h or CD1h transplanted kidneys (Figure 2A). These data suggest that massive RCC in the IS disappears by 2 days after transplantation in CD0h or CD1h kidneys, but not in CD2h kidneys. Quantification of red cell trapping in the four regions of transplanted kidney tissues showed that CD1h kidneys had smaller areas of RCC in the cortex (CTX), outer stripe of the outer medulla (OS), and inner medulla (IM) areas than CD0h, whereas these areas had increased in CD2h kidneys (Figure 2B). Changes in the area of RCC were very different in the IM than in the other three regions of kidney tissue we examined. In CD2h kidneys, they kept increasing, finally culminating in massive quantities. These data suggest that RCC behaves very differently in the IS than in the other three regions.

### Mechanisms of RCC trapping in CD kidney grafts

**Figure 3:**
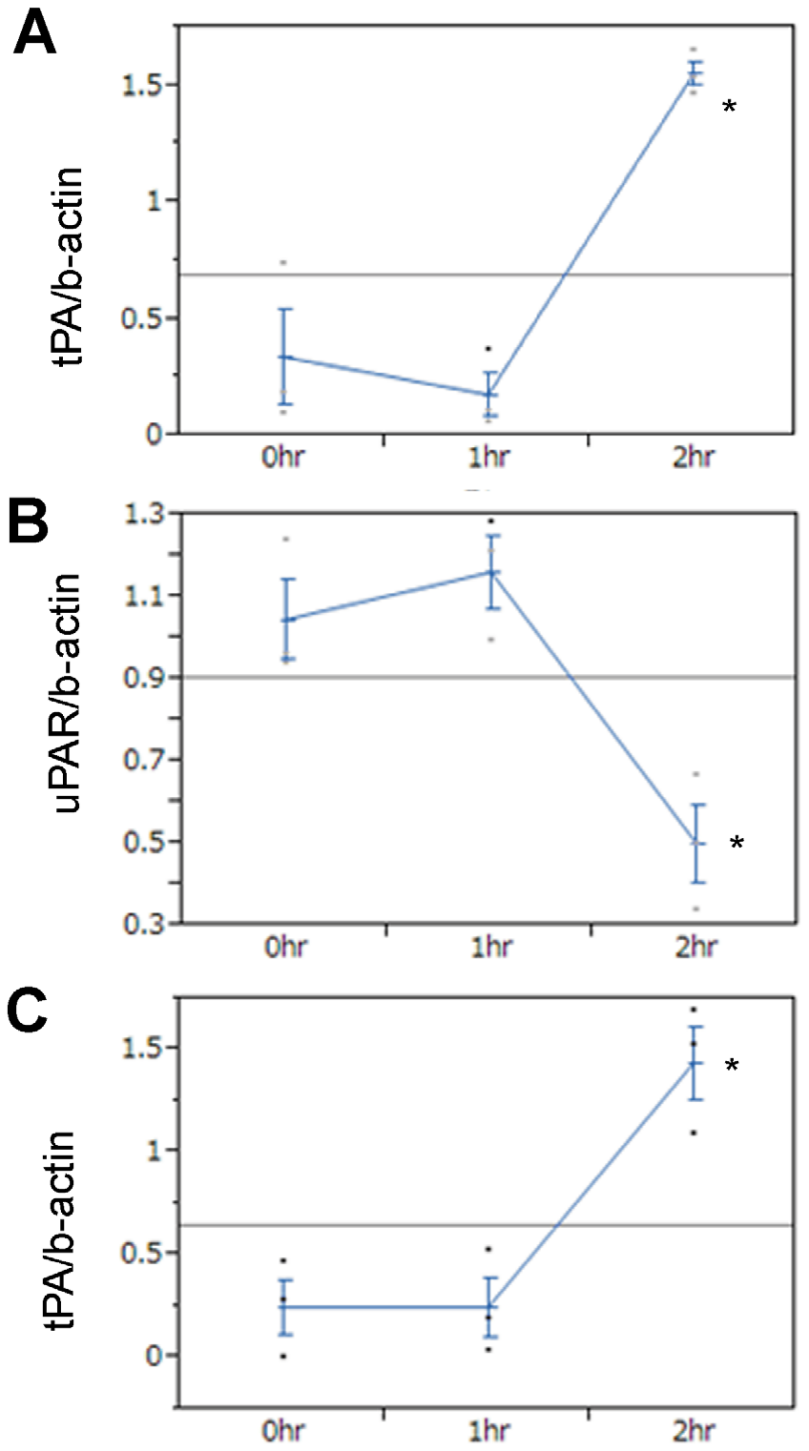
Gene expression profiles of transplanted cardiac death (CD) kidney graft cortexes and medullas. At two days after kidney transplantation, each animal was sacrificed and its transplanted kidney was harvested. By using isolated mRNA from transplanted kidneys, gene expression profiles were investigated. (A) tPA/beta-actin in cortex. (B) uPAR/beta-actin in cortex. (C) tPA/beta-actin in medulla. The each graph shows that respective gene expression was significantly increased in CD2h, compared with CD0h. *, p<0.05 vs CD0h unpaired t-test.

To investigate the mechanism of massive RCC generation in only the IS of CD2h transplanted kidneys, we examined the gene expression profiles (TGF-β, α-SMA, HIF-1α, MCP-1, HO-1, HSP-70, IL-6, KIM-1, tPA, uPA, uPAR, PAI-1) of CD kidney cortexes and medullas. Compared with CD0h and CD1h kidneys, CD2h kidneys showed greatly increased gene expression of t-PA, but not u-PA, in transplanted cortexes and medullas, suggesting that t-PA mRNA expression induces impaired circulation and RCC in the IS (Figure 3A, C). Although the blood coagulation system is not involved in generation of this congestion [7], coagulation may be involved in the maintenance or amplification of RCC long after reperfusion. These data are compatible with the data on RCC in transplanted CD kidney grafts (Figure 2). Of the other candidate genes, the only significant change we identified in CD2h tissue was in uPAR in medullas (Figure 3B, Figure S1).

### Massive trapping of red blood cells in CD kidneys before transplantation

**Figure 4:**
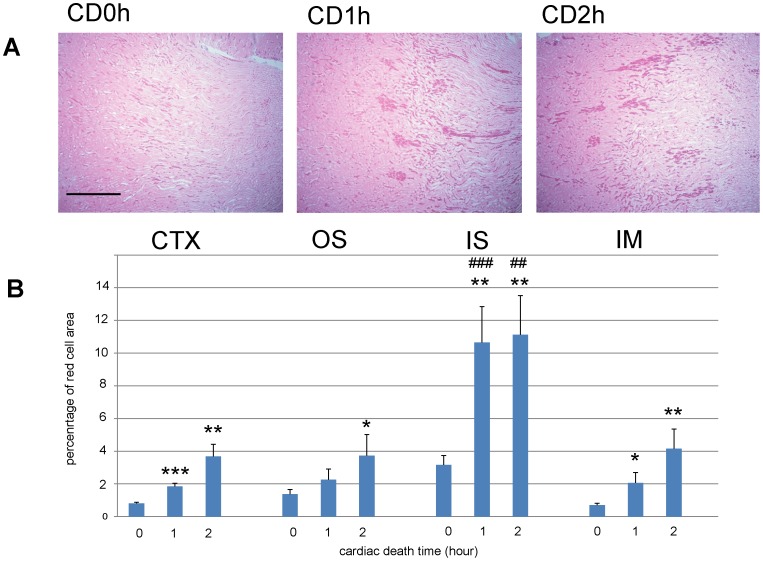
Before transplantation, red cell congestion (RCC) were equally observed in IS of 1h cardiac death (CD1h) and CD2h kidney grafts. Eosin-stained photomicrographs before transplantation. These images show the transition area between the outer and inner stripes of the outer medulla of CD kidney grafts. The large RCC were observed in inner stripe of the outer medulla (IS) of CD1h and CD2h kidney grafts in the same fashion. Scale bar: 0.5 mm. (A) Quantification of red cell area congestion in various regions of CD kidney grafts. The massive RCCs were equally measured in IS of CD1h and CD2h kidney grafts. *, p<0.05 vs CD0h;**, p<0.01; ***, p<0.001 vs CD0h unpaired t-test; ###, p<0.001; ##, p<0.01, one-way ANOVA and Tukey’s test.

When we examined kidney tissue specimens from CD donors 0–2 h after cardiac arrest and before transplantation, we found that CD1h and CD2h, but not CD0h, kidneys contained massive trapped red cells, but only in the IS (Figure 4A). Quantification of red cell trapping in four regions of the transplanted kidneys revealed that the areas of RCC gradually increased in the CTX, OS, and IM, whereas in the IS they had increased massively at CD1h and this was still true at CD2h (Figure 4B). RCC areas in the IS were significantly larger in CD1h and CD2h than in CD0h kidneys.

### Non-invasive assessment of trapped red cells in kidney grafts using blood oxygen level-dependent (BOLD) MRI

**Figure 5:**
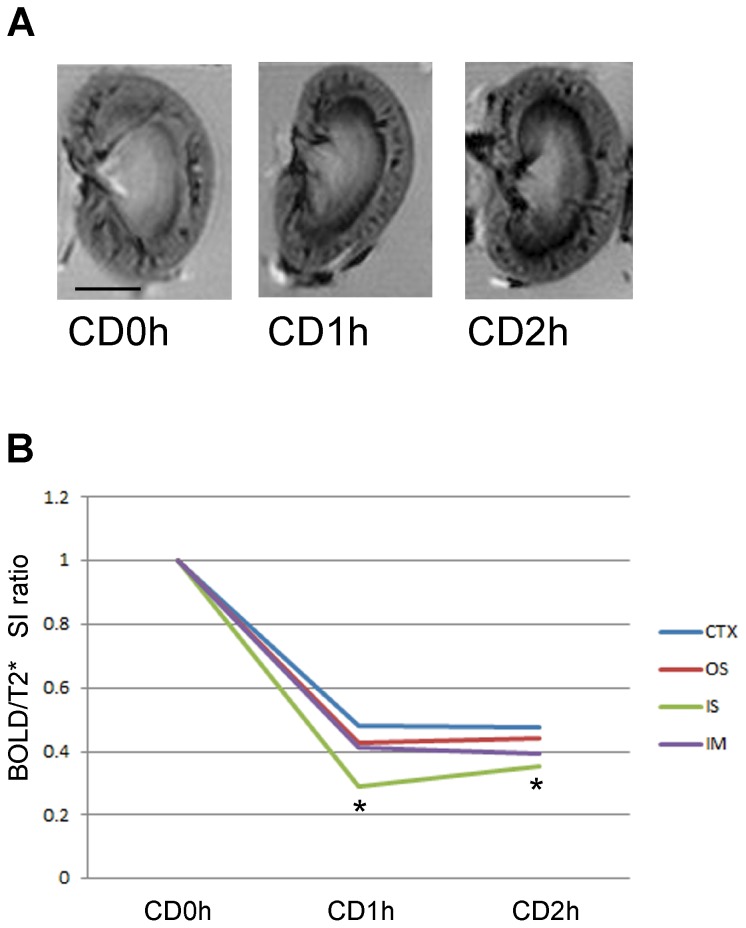
T_2_* weighted MRI successfully detect massive red cell congestion (RCC) in 1h cardiac death (CD1h) and CD2h kidney grafts. (A) T_2_* weighted images of CD 0, 1 and 2 h kidney grafts before transplantation. The large decreases of signal intensity in the T_2_* weighted images were observed along IS of CD1h and CD2h kidney grafts. Scale bar: 0.5 cm. (B) T_2_* signal intensity (SI) ratio of CD 0, 1 and 2 h kidney grafts before transplantation. T_2_* SI ratio: SI(CDnh)/mean SI(CD0h), n = 0,1,2. T_2_* SI ratio of IS of CD1h and CD2h kidney grafts were significantly reduced, compared with other parts of kidney in the same CD kidney tissues. *, p<0.05, one-way ANOVA and Tukey’s test.

Next, we used BOLD MRI to non-invasively assess the degree to which red cells are trapped in kidneys from CD donors. BOLD MRI can detect trapped red cells by sensing deoxygenated hemoglobin, which is an indicator of the degree of ischemia. We harvested the left kidneys from three CD donors (0, 1, 2 h) and assessed them with BOLD MRI. T_2_* weighted MR images of the CD kidneys provided images of trapped blood cells in the IS of the outer medulla in CD1h and CD2h kidney grafts, concordant with the histological findings in tissue sections (Figures 4A and 5A). In addition, we detected decreases in signal intensity of T_2_* weighted MRI images in all four regions of kidney tissue studied (Table 1) and were able to identify that RCC in the IS of CD1h or CD2h kidney grafts had the largest decrease in signal intensity ratio of T_2_* weighted MRI (Figure 5B). A further point of interest is that incubation in UW solution for 3 h caused a decrease in the signal intensity of BOLD MRI of CD0h kidney grafts, whereas the signal intensity of CD1h kidneys did not change and the signal intensity of CD2h kidneys increased in the CTX and OS (Table 2). Therefore, changes in signal intensity of T_2_* weighted MRI may correlate with viability of kidney grafts. These data suggest that BOLD MRI (T_2_* weighted MRI) can detect areas of trapped blood red cells in graft kidneys from CD donors non-invasively even when organ preserving solutions (UW solution) have been used.

**Table 1:** Average T_2_* values (*n* = 3) in renal compartments at specified times after cardiac death (CD).

Renal compartments	T_2_* CD0h±SD [10^−3^ s]	T_2_* CD1h±SD [10^−3^ s] (p value)	T_2_* CD2h±SD [10^−3^ s] (p value)
CTX	146.7±10.8	70.4±1.4 (0.0003)	69.9±1.3 (0.0003)
OS	133.5±3.8	56.8±5.2 (<0.0001)	58.7±2.1 (<0.0001)
IS	146.1±14.1	42.3±5.8 (0.0003)	51.3±0.6 (0.0003)
IM	218.1±10.3	90.1±4.0 (<0.0001)	84.8±3.6 (<0.0001)

CTX, cortex; IM, inner medulla; IS, inner stripe of outer medulla; OS, outer stripe of outer medulla.

**Table 2:** Average T_2_* values (*n* = 3) in renal compartments before and after incubation in University of Wisconsin (UW) solution.

Renal compartment	T_2_*CD0h before UW±SD [10^−3^ s]	T_2_*CD0h after UW±SD [10^−3^ s]	p value
CTX	146.7±10.8	87.3±7.8	0.0021
OS	133.5±3.8	81.4±4.9	0.0044
IS	146.1±14.1	87.9±9.0	0.0026
IM	218.1±10.3	137.6±6.5	0.0008

Renal compartment	T_2_*CD1h before UW±SD [10^−3^ s]	T_2_*CD1h after UW±SD [10^−3^ s]	p value
CTX	70.4±1.4	70.1±1.4	0.8095
OS	56.8±5.2	58.0±3.4	0.6461
IS	42.3±5.8	42.1±3.0	0.9227
IM	90.1±4.0	91.3±3.9	0.0861

Renal compartment	T_2_*CD2h before UW±SD [10^−3^ s]	T_2_*CD2h after UW±SD [10^−3^ s]	p value
CTX	69.9±1.3	78.0±0.9	0.0251
OS	58.7±2.1	69.7±1.5	0.0298
IS	51.3±0.6	55.0±3.1	0.1597
IM	86.0±9.6	94.9±11.4	0.4096

CTX, cortex; IM, inner medulla; IS, inner stripe of outer medulla; OS, outer stripe of outer medulla.

### Identification of increased apparent diffusion coefficient (ADC) in CD kidney grafts by diffusion weighted (DW) MRI

**Figure 6:**
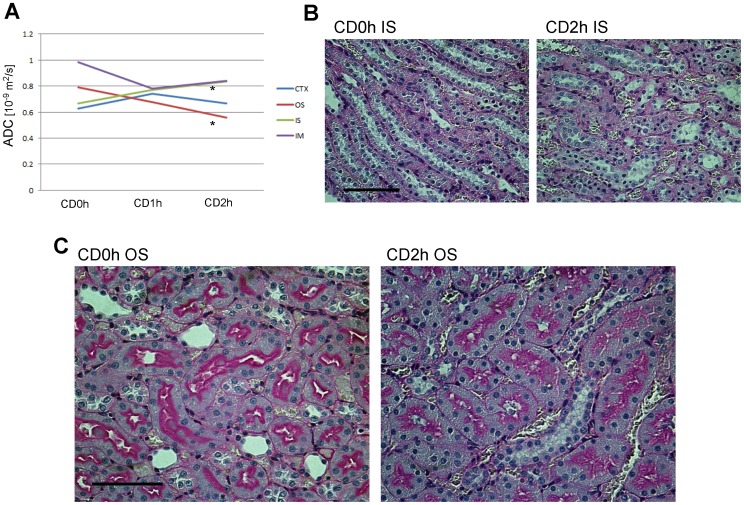
diffusion weighted (DW) MRI successfully detected different kinds of edema in outer stripe of the outer medulla (OS) and inner stripe of the outer medulla (IS). (A) Graph showing apparent diffusion coefficient (ADC) values in each region of cardiac death (CD) kidney grafts. In CD2h kidney grafts, ADC values in OS and IS were significantly decreased and increased respectively, compared with CD0h kidney grafts. *, p<0.05 vs CD0h, unpaired t-test. (B) Periodic acid-Schiff (PAS)-stained photomicrographs of OS of CD0h and CD2h kidney grafts. In OS of CD2h kidney grafts, more interstitial edema was observed than in CD1h kidney grafts. Scale bar: 100 µm. (C) PAS-stained photomicrographs of IS in CD0h and CD2h kidney grafts. In IS of CD2h kidney grafts, more intracellular edema was observed than in CD1h kidney grafts. Scale bar: 50 µm.

In this study, we identified massive RCC in the IS of both CD1h and CD2h kidney grafts before transplantation. However, 2 days after transplantation, RCC persisted only in the IS of CD2h kidney grafts, whereas it had disappeared from the IS of CD1h kidneys. Because it is speculated that dramatic changes in capillary permeability occur during generation of RCC in the IS of ischemic kidneys, we tried to identify what determines the observed differences in RCC in the IS by using diffusion MRI to examine how water molecule behavior changes in CD renal tissues. We harvested the left kidneys from three CD donors (0, 1, 2 h) and assessed them by diffusion MRI and calculated ADC values (Figure 1). We found that only in the IS of the outer medullas were ADC values significantly increased 2 h after transplantation, whereas these values were decreased after 2 h in the OS of the outer medullas and statistically unchanged in the CTX and IM (Table 3 and Figure 6A). These data suggest that only the IS of the outer medullas have interstitial edema, whereas the OS have cellular edema, to a degree that is detectable by diffusion MRI. Consistent with these ADC results, the OS in CD2h kidney grafts had more interstitial enlarged areas than CD0h kidney grafts (Figure 6C) and the IS in CD2h kidney grafts more edematous tubule cells than CD0h kidney grafts (Figure 6B). With 3 h incubation in UW solution, the ADC values had a tendency to decrease, particularly in the medullas (Table 4).

**Table 3:** Average apparent diffusion coefficient (ADC) values (*n* = 3) in renal compartments at specified times after cardiac death (CD).

Renal compartment	ADC CD0h±SD [10^−9^ m^2^/s]	ADC CD1h±SD [10^−9^ m^2^/s] (p value)	ADC CD2h±SD [10^−9^ m^2^/s] (p value)
CTX	0.626±0.043	0.743±0.070 (0.2157)	0.667±0.054 (0.3588)
OS	0.793±0.066	0.676±0.120 (0.2118)	0.558±0.068 (0.0127)
IS	0.665±0.073	0.773±0.067 (0.2274)	0.833±0.030 (0.0213)
IM	0.985±0.029	0.783±0.097 (0.5582)	0.840±0.120 (0.1183)

CTX, cortex; IM, inner medulla; IS, inner stripe of outer medulla; OS, outer stripe of outer medulla.

**Table 4:** Average ADC values (*n* = 3) in renal compartments before and after incubation in University of Wisconsin (UW) solution.

Renal compartment	ADC CD0h before UW±SD [10^−9^ m^2^/s]	ADC CD0h after UW±SD [10^−9^ m^2^/s]	p value
CTX	0.626±0.043	0.677±0.033	0.3582
OS	0.793±0.066	0.750±0.057	0.0325
IS	0.665±0.073	0.517±0.013	0.0495
IM	0.985±0.029	0.871±0.011	0.0095

Renal compartment	ADC CD1h before UW±SD [10^−9^ m^2^/s]	ADC CD1h after UW±SD [10^−9^ m^2^/s]	p value
CTX	0.743±0.070	0.660±0.157	0.2422
OS	0.676±0.120	0.470±0.081	0.1241
IS	0.773±0.067	0.607±0.056	0.0496
IM	0.783±0.097	0.630±0.085	0.0164

Renal compartment	ADC CD2h before UW±SD [10^−9^ m^2^/s]	ADC CD2h after UW±SD [10^−9^ m^2^/s]	p value
CTX	0.667±0.054	0.609±0.012	0.1842
OS	0.558±0.068	0.501±0.087	0.4284
IS	0.833±0.030	0.660±0.083	0.0305
IM	0.840±0.120	0.723±0.160	0.0365

CTX, cortex; IM, inner medulla; IS, inner stripe of outer medulla; OS, outer stripe of outer medulla.

### Effects of University of Wisconsin (UW) solution perfusion on CD kidney grafts detected by BOLD and DW MRI

**Figure 7:**
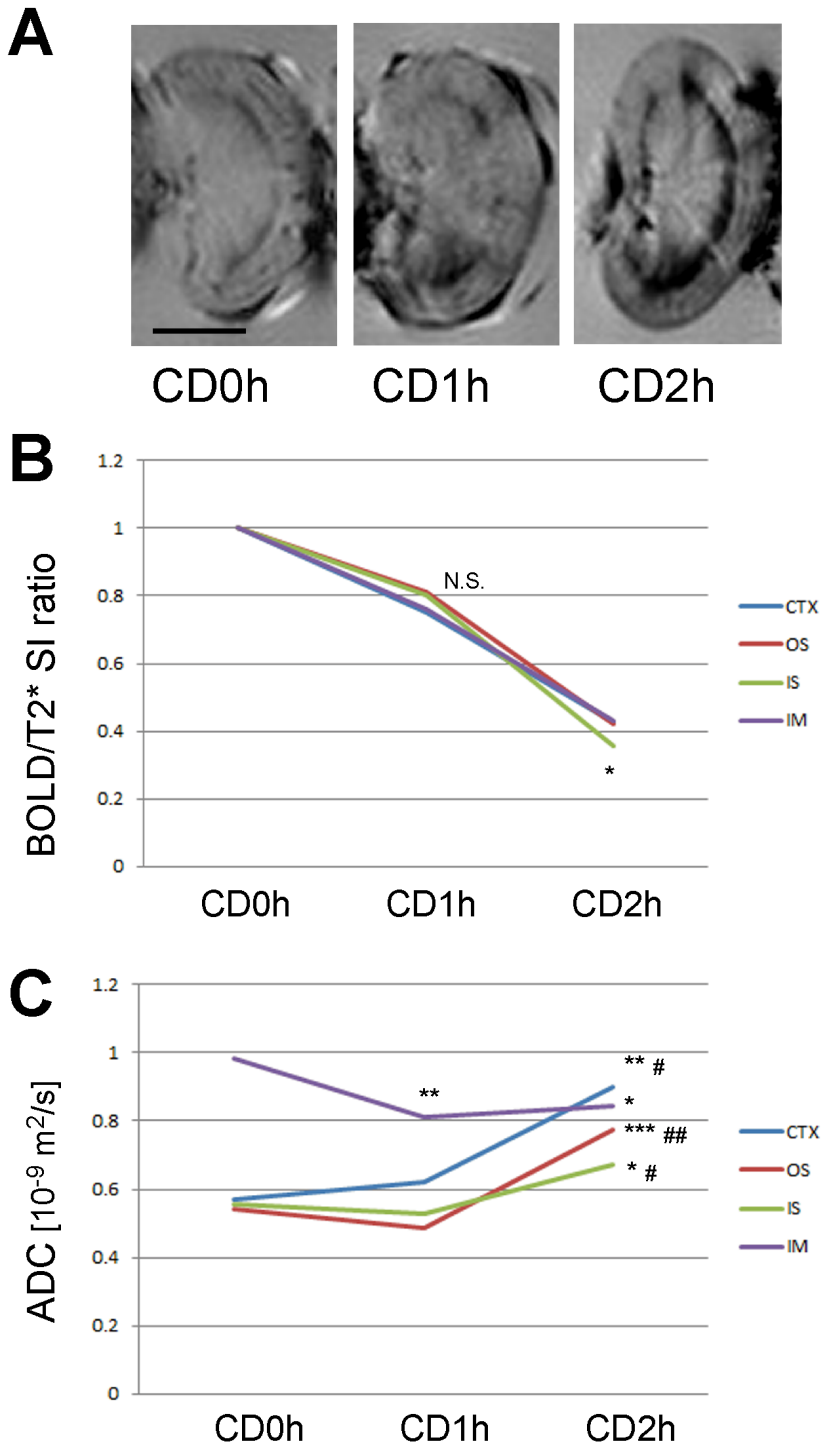
blood oxygen level-dependent magnetic resonance imaging (BOLD) and diffusion weighted (DW) MRI successfully detected University of Wisconsin (UW) solution perfused 2h cardiac death (CD2h) kidney grafts. BOLD/T_2_* weighted images of CD 0, 1 and 2 h kidney grafts after perfusion with UW solution. The RCC was partially washed out from inner stripe of the outer medulla (IS) of CD1h, but almost not from IS of CD2h kidney grafts. Scale bar: 0.5 cm. (A) T_2_* signal intensity (SI) ratios of CD 0, 1 and 2 h kidney grafts after perfusion with UW solution. T_2_* SI ratio: SI(CDnh)/mean SI(CD0h), n = 0,1,2. T_2_* SI ratio of IS of CD1h kidney grafts was not significantly reduced, whereas T_2_* SI ratio of IS of CD2h kidney grafts was significantly reduced, compared with other parts of kidney in the same CD kidney tissues, suggesting RCC in IS of CD1h kidney grafts was partially washed out by perfusion. *, p<0.05, one-way ANOVA and Tukey’s test. (B) apparent diffusion coefficient (ADC) values of CD kidney grafts after perfusion with UW solution. ADC values in cortex (CTX), outer stripe of the outer medulla (OS) and IS of CD2h kidney grafts were significantly increased, compared with CD0h and CD1h kidney grafts, suggesting that UW solution perfusion made clearer the difference between CD1h and CD2h kidney tissue damages than before the perfusion.*, p<0.05 vs CD0h; #, p< unpaired t-test.

Thus far, this study has focused on MRI study of kidney grafts that we had not perfused after harvest, because we performed our previous kidney transplantations without perfusion [10]. However, because CD kidney grafts are conventionally perfused with UW solution in real clinical settings, we examined perfused CD kidney grafts with BOLD and DW MRI. In the BOLD images, RCC was partially washed out from the IS of CD1h kidneys. However, it was not washed out from the IS of CD2h kidneys (Figure 7A). Histological assessment of tissue specimens confirmed these BOLD image results (Figure S2). Consistent with the BOLD images, we detected decreases in signal intensity of T_2_* weighted MRI in all four regions of the kidneys at CD1h and CD2h (Table 5), but identified that RCC only in the IS of CD2h kidney grafts had the largest decreases in signal intensity ratio of T_2_* weighted MRI (Figure 7B). DW MRI measurements showed that ADC values in the CTX, OS and IS segments increased greatly in CD2h grafts, whereas they decreased in the IM of CD1h grafts (Figure 7C, Table 6). Histological examination of tissue specimens confirmed that these ADC increases and decreases represented interstitial and cellular edema, respectively (Figure S2). Subsequent kidney perfusion with UW solution elucidated that tissue injuries correlated with decreases in signal intensity ratio of T_2_* in the IS and increases in ADC values in the CTX, OS and IS.

**Table 5:** Average T_2_* values (*n* = 3) in renal compartments at specified times after cardiac death (CD) after perfusion with University of Wisconsin (UW) solution.

Renal compartment	T_2_* CD0h ± SD[10^−3^ s]	T_2_* CD1h ± SD[10^−3^ s] (p value)	T_2_* CD2h ± SD[10^−3^ s] (p value)
CTX	187.3±6.6	140.7±3.0 (0.0004)	80.2±5.7 (<0.0001)
OS	182.9±13.1	148.5±1.0 (0.0107)	77.1±5.0 (0.0002)
IS	177.9±16.5	143.0±6.6 (0.0269)	63.6±2.1 (0.0003)
IM	222.4±9.3	168.7±12.4 (0.0039)	95.9±4.0 (<0.0001)

CTX, cortex; IM, inner medulla; IS, inner stripe of outer medulla; OS, outer stripe of outer medulla.

**Table 6:** Average apparent diffusion coefficient (ADC) values (*n* = 3) in renal compartments at specified times after cardiac death (CD) after perfusion with University of Wisconsin (UW) solution.

Renal compartment	ADC CD0h ± SD [10^−9^ m^2^/s]	ADC CD1h ± SD [10^−9^ m^2^/s] (p value)	ADC CD2h ± SD [10^−9^ m^2^/s] (p value)
CTX	0.568±0.060	0.621±0.091 (0.4498)	0.897±0.094 (0.0069)
OS	0.543±0.028	0.486±0.068 (0.2484)	0.774±0.008 (0.0002)
IS	0.557±0.011	0.528±0.054 (0.4060)	0.671±0.053 (0.0213)
IM	0.983±0.013	0.810±0.060 (0.0079)	0.843±0.064 (0.0216)

CTX, cortex; IM, inner medulla; IS, inner stripe of outer medulla; OS, outer stripe of outer medulla.

## Discussion

In the present study, we demonstrated that CD2h, but not CD0h or CD1h kidneys, exhibit massive RCC in the IS 2 days after transplantation. Previously, we showed that CD0h and CD1h kidney grafts function after transplantation, but that bilaterally nephrectomized rats receiving CD2h kidneys developed high creatinine and BUN concentrations and did not survive more than 5 days after transplantation, suggesting the transplanted CD2h kidney grafts did not function [10]. We reasoned that the amount of RCC persisting in the IS 2 days after transplantation might predict graft function. Although CD1h and CD2h kidney grafts developed RCC in the IS equally before transplantation, RCC remained only in this region of CD2h kidney grafts 2 days after transplantation.

Consistent with the histological observations, tPA gene expression was increased in the CTX and medullas of transplanted CD2h kidney grafts. tPA, and not uPA, reportedly acts predominantly in blood vessels in a paracrine manner [21]. We confirmed no significant changes in expression of other genes in CD2h tissues, with the exception of uPAR in the medullas. Although we do not know why uPAR gene expression was reduced in CD2h kidney medullas, ischemia/reperfusion injury is reportedly ameliorated in uPAR knockout mouse [22], suggesting that this change in expression uPAR gene is logical.

We have shown that detection of prolonged RCC in the IS by BOLD and diffusion MRI can predict graft injuries in transplanted kidneys. RCC in the IS indicates established ischemic damage in kidney tissue, which predicts the long-term outcome of transplants [20]. We used the non-invasive technique of BOLD MRI to identify massive RCC in the IS of CD kidney grafts before transplantation. We visualized massive RCC in the IS of CD1h and CD2h kidney grafts by T_2_* weighted MR images and identified the same phenomena as reduced signal intensity ratios of T_2_*WI. Because we have shown that massive RCC in the IS after renal transplantation leads to primary non-function of the transplanted kidney, RCC in the IS after transplantation could be another predictor of renal graft outcome. In addition, we observed increases in signal intensity of T_2_*WI in the CTX and OS of CD2h grafts after 3 h incubation in UW solution. These phenomena may serve as other types of markers of viability of CD kidney grafts. However, it is largely unknown why signal intensity of T_2_*WI in CTX and OS of CD2h grafts increase after 3 h incubation in UW solution.

**Figure 8:**
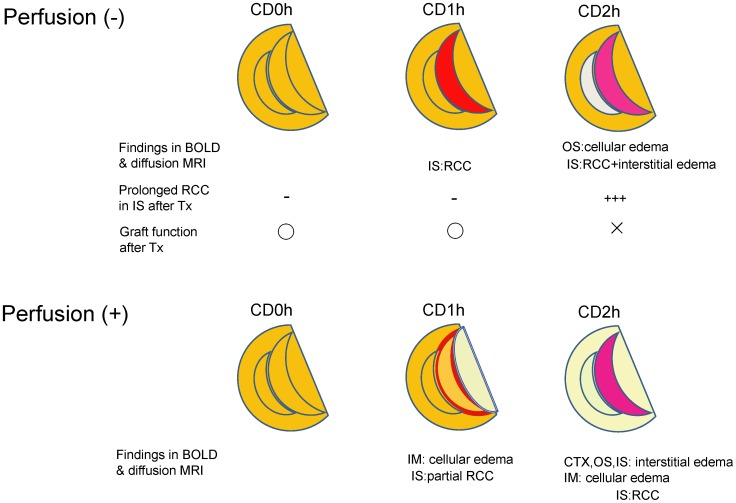
Schematic representation of cardiac death (CD) kidney graft tissue damage non-invasively identified by blood oxygen level-dependent magnetic resonance imaging (BOLD) and diffusion weighted (DW) MRI. In IS of CD1h and CD2h kidney grafts before transplantation, red cell congestion (RCC) was observed almost equally. But the RCC in IS of CD1h kidney grafts disappeared after transplantation, whereas RCC in CD2h did not. As one of causes for these differences, two different kinds of edemas were detected in OS and IS of CD2h kidney tissue. This remained RCC in IS may cause poor outcomes of transplanted CD kidney grafts. The UW solution perfusion made clearer the difference between CD1h and CD2h kidney tissue damages than before the perfusion. CTX, cortex; IM, inner medulla; IS, inner stripe of outer medulla; OS, outer stripe of outer medulla; RCC, red cell congestion; Tx, transplantation. About “Prolonged RCC in IS after Tx”, - means “not observed” and +++ means “clearly observed”. About “Graft function after Tx”, O means “functioning” and × means “not functioning”.

We used diffusion MRI and calculation of ADC in CD graft kidney tissues to investigate the reasons for the observed differences in sustained RCC in CD kidneys. We demonstrated that CD2h kidney grafts have decreased ADC in the OS, but increased ADC in the IS. Because increased and decreased ADC represent interstitial and cellular edema, respectively [17], the changes in ADC values identified by diffusion MRI suggest that there is interstitial edema in the IS of CD2h kidney grafts. This is consistent with the observation that interstitial edema is a common finding in human biopsy specimens of acute kidney injury [23]. In addition, tubular cellular edema in the OS, which decreased ADC in this region suggests, may inhibit erythrocytes from coming out of the medulla back up to the cortex, resulting in a vicious cycle of graft dysfunction [24] (Figure 8).

Because graft perfusion after harvest is conventionally performed prior to human transplantation, we examined perfused as well as non-perfused kidney grafts by MRI. In the case of graft kidney tissues without perfusion, the standard deviation shows that values between CD1h and CD2h can overlap (Table 3), and although only CD2hvalues are significantly different from 0h, the absence of sharp discrimination could be problematic as a clinical marker. Surprisingly, however, UW solution perfusion enhanced the tissue differences between CD1h and CD2h grafts according to both BOLD and DW MRI, suggesting that this MRI method is also applicable to actual clinical settings.

## Conclusion

BOLD and diffusion MRI are readily available non-invasive tools for evaluating CD kidney graft tissue damage and may predict the outcome of transplanted CD kidney grafts. It also revealed that the conventional perfusion of CD kidney grafts with an organ preserving solution made kidney grafts damage more enhanced, suggesting that BOLD and diffusion MRI can be applied in real clinical settings.

## Supporting Information

Figure S1 **Gene expression profiles of transplanted CD kidney grafts**. In CTX, HO-1, HSP-70, TGF-b, KIM-1, IL-6, HIF-1α, MCP-1, uPA, and α-SMA gene expressions were quantified. HIF-1αgene expression was significantly expressed in CD1h kidney grafts, compared with CD0h. In MD, HO-1, HSP-70, TGF-b, KIM-1, IL-6, HIF-1α, MCP-1, uPA, uPAR, and α-SMA gene expressions were investigated. No significant difference was detected between CD kidney grafts. CTX, cortex; MD, medulla. *, p<0.05 vs CD0h unpaired t-test.
(TIF)

Figure S2 **PAS**-**stained photomicrographs** **of perfused CD0h, CD1h and CD2h kidney grafts**. In perfused CD2h kidney grafts, interstitial edema was observed in (A) Cortex Outer stripe of outer medulla. (Scale bar: 100 µm), (B) Outer stripe of outer medulla, and (C) Inner stripe of outer medulla. (D) Inner medulla in CD1h and CD2h kidney grafts, intracellular edema was observed. Scale bar: 50 µm.
(TIF)
