# Supplementary figures and images for: Non-Invasive Magnetic Resonance Imaging in Rats for Prediction of the Fate of Grafted Kidneys from Cardiac Death Donors
Source: PLoS One. 2013 May 7;8(5):e63573. doi: 10.1371/journal.pone.0063573 (PMC3647057; doi:10.1371/journal.pone.0063573)

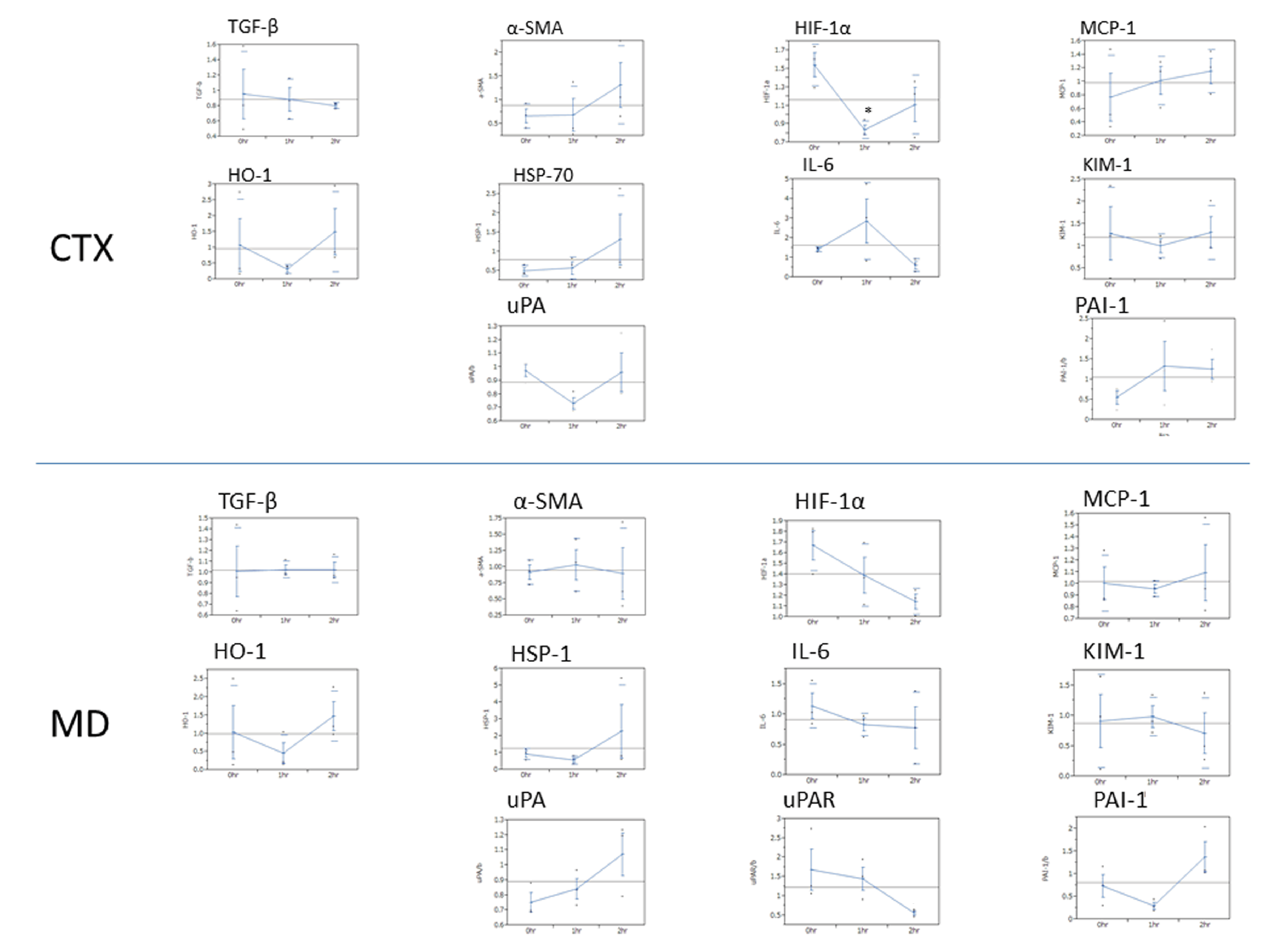

Supplement: Figure S1 — Gene expression profiles of transplanted CD kidney grafts. In CTX, HO-1, HSP-70, TGF-b, KIM-1, IL-6, HIF-1α, MCP-1, uPA, and α-SMA gene expressions were quantified. HIF-1αgene expression was significantly expressed in CD1h kidney grafts, compared with CD0h. In MD, HO-1, HSP-70, TGF-b, KIM-1, IL-6, HIF-1α, MCP-1, uPA, uPAR, and α-SMA gene expressions were investigated. No significant difference was detected between CD kidney grafts. CTX, cortex; MD, medulla. *, p<0.05 vs CD0h unpaired t-test. (TIF) [file pone.0063573.s001.tif]

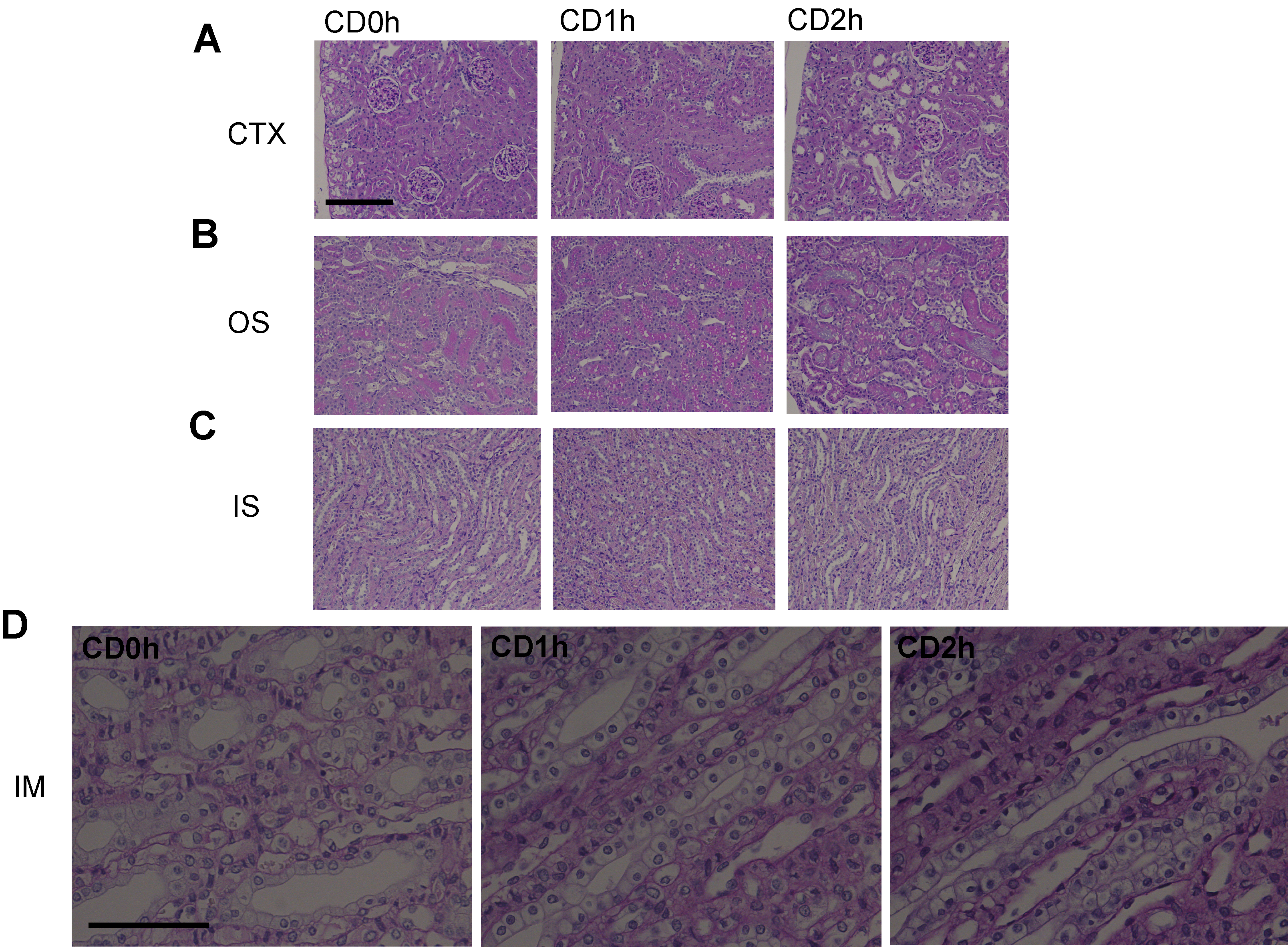

Supplement: Figure S2 — PAS-stained photomicrographs of perfused CD0h, CD1h and CD2h kidney grafts. In perfused CD2h kidney grafts, interstitial edema was observed in (A) Cortex Outer stripe of outer medulla. (Scale bar: 100 µm), (B) Outer stripe of outer medulla, and (C) Inner stripe of outer medulla. (D) Inner medulla in CD1h and CD2h kidney grafts, intracellular edema was observed. Scale bar: 50 µm. (TIF) [file pone.0063573.s002.tif]
